# Effectiveness of strategies to increase the validity of findings from association studies: size vs. replication

**DOI:** 10.1186/1471-2288-10-47

**Published:** 2010-05-28

**Authors:** Rolf Weitkunat, Etienne Kaelin, Grégory Vuillaume, Gerd Kallischnigg

**Affiliations:** 1 Department of Biostatistics & Epidemiology, R&D, Philip Morris International, Neuchâtel, Switzerland

## Abstract

**Background:**

The capacity of multiple comparisons to produce false positive findings in genetic association studies is abundantly clear. To address this issue, the concept of false positive report probability (FPRP) measures "the probability of no true association between a genetic variant and disease given a statistically significant finding". This concept involves the notion of prior probability of an association between a genetic variant and a disease, making it difficult to achieve acceptable levels for the FPRP when the prior probability is low. Increasing the sample size is of limited efficiency to improve the situation.

**Methods:**

To further clarify this problem, the concept of true report probability (TRP) is introduced by analogy to the positive predictive value (PPV) of diagnostic testing. The approach is extended to consider the effects of replication studies. The formula for the TRP after k replication studies is mathematically derived and shown to be only dependent on prior probability, alpha, power, and number of replication studies.

**Results:**

Case-control association studies are used to illustrate the TRP concept for replication strategies. Based on power considerations, a relationship is derived between TRP after k replication studies and sample size of each individual study. That relationship enables study designers optimization of study plans. Further, it is demonstrated that replication is efficient in increasing the TRP even in the case of low prior probability of an association and without requiring very large sample sizes for each individual study.

**Conclusions:**

True report probability is a comprehensive and straightforward concept for assessing the validity of positive statistical testing results in association studies. By its extension to replication strategies it can be demonstrated in a transparent manner that replication is highly effective in distinguishing spurious from true associations. Based on the generalized TRP method for replication designs, optimal research strategy and sample size planning become possible.

## Background

The advent of the "omics" revolution caused molecular epidemiologists to attempt to rapidly identify as many associations between genetic variants (most often single nucleotide polymorphisms [SNPs]) and diseases as possible. In fact, discoveries were announced at impressive rates with respect to various diseases, particularly with the introduction of high-throughput genotyping technologies and genome-wide association studies. Hoover ([1], p. 14) speaks of a "blizzard of positive findings". This came as no surprise to statisticians, as it is not uncommon in "omics" studies to set a particular α-level and call any association with a p-value below α significant [2].

It soon became increasingly clear, however, that the vast majority of these "discoveries" could not be replicated in subsequent studies. Ioannidis [3] pointed out that the findings from single association studies (which are usually adaptations of the case-control design) constitute "tentative knowledge" and must be interpreted with exceptional caution. Initial findings often overestimated the true effect size [4,5], and reviews have shown that "an alarming proportion" ([6], p. 421) of initial findings of associations between genetic variants and diseases were irreproducible [7,8]. As Pharoah *et al*. [9] noted, the most likely explanation for this phenomenon was "that most initial reports are false positive, and the most common reason for this is simply chance/type I error, exacerbated by publication bias" (p. 852).

In the context of identifying associations between genetic variants and diseases and to overcome the problem of having too many false positives, Wacholder *et al*. [10] introduced the concept of false positive report probability (FPRP). The FPRP measures "the probability of no true association between a genetic variant and disease given a statistically significant finding" and can be used to decide whether a finding is deserving attention or not. The FPRP depends on the prior probability of an association between the genetic variant and the disease, on the α-level, and on the power of the statistical test. It is important to mention that if the prior probability is low, the FPRP cannot reach acceptable levels, even in the case of very high power of the statistical test.

To further address this point, the concept of True Report Probability (TRP) for statistical hypothesis tests is introduced by analogy to the positive predictive value (PPV) of a diagnostic test. The TRP concept is extended to combine the results of several studies to provide a method for maximizing the probability of "significant" associations between a genetic variant and a disease being true (even in the case of low prior probability of an association, and without requiring extreme sample sizes). The framework can be used by researchers to design more conclusive research strategies.

## Methods and Results

### Analogy between Diagnostic and Statistical Testing

Diagnostic testing is used to separate cases from non-cases in a given population. The set-up is usually summarized by a four-fold table where each cell represents the absolute number of individuals for the different combinations of outcome (disease D+ vs. no disease D-) and test result (positive T+ vs. negative T-) (see Additional file 1: Appendix A, Table A1). Sensitivity (proportion of diseased individuals who tested positive) and specificity (proportion of healthy individuals who tested negative) are the two key properties of a diagnostic test. Moreover, the positive predictive value of a diagnostic test is defined as the probability of the disease given a positive test result.

The PPV is related to sensitivity and specificity through Bayes' theorem:(1)

where P(D+) is the prevalence of the disease (which may be derived from cross-sectional studies), Sens the sensitivity, and Spec the specificity.

If the disease prevalence is low (around 0.001), the PPV obtained by applying one diagnostic test is low (<0.1), even for high values of sensitivity and specificity. However, the PPV can be increased by using more than one diagnostic test successively in individuals who tested positive in previous tests. This is illustrated by the HIV example using the Elisa and Western blot tests successively (see Additional file 1: Appendix A, Tables A2 and A3).

The sensitivity and specificity of the Elisa and Western blot tests is in this example assumed to be 0.95 and 0.99, respectively, and the prevalence of HIV in a given population to be 0.001. If only the Elisa test is performed in the initial population of 1'000'000 individuals, the PPV of this test is as low as 0.087, whereas the number of individuals who tested positive is 10'940. If subsequently the Western blot test is applied in Elisa-positive individuals, the HIV prevalence in this sub-population equals the positive predictive value of the Elisa test (0.087), and the PPV increases to 0.9. The example illustrates the benefits of "replication" and its impact on the confidence in a confirmed positive finding.

### True Report Probability

The concepts of diagnostic testing can be adapted to investigate associations between a genetic variant and a disease. The null hypothesis (H_0_) of the statistical test is that there is no association between a specific genetic variant and a disease, while the alternative hypothesis (H_1_) states an association (see Table 1, which gives a complete overview of the correspondence between diagnostic and statistical testing).

**Table 1:** Correspondence of characteristics of diagnostic and statistical testing.

Diagnostic test	Statistical test
Presence of disease (D+)	Association (H_1 _true)
Absence of disease (D-)	No association (H_0 _true)
Positive result (T+)	Rejecting H_0_
Negative result (T-)	Retaining H_0_
Sensitivity = P(T+ | D+)	Power = P(H_0 _rejected | H_1 _true) = 1 - β
1 - Specificity = 1 - P(T- | D-) = P(T+ | D-)	Significance level = P(H_0 _rejected | H_0 _true) = α
Prevalence of disease = P(D+)	Prior probability of association = P(H_1 _true) = π
Positive predictive value = PPV = P(D+ | T+)	True report probability = TRP = P(H_1 _true | H_0 _rejected) = 1 - P(H_0 _true | H_0 _rejected)

The PPV of diagnostic testing corresponds to the true report probability in statistical testing, i.e. the probability of a true association, given a statistically significant result:(2)

where π is the prior probability of an association. Estimation of π is context-dependent and can be based on previous studies (Dubé *et al*., [11]) or on subject-matter considerations (Ziegler *et al*., [12]; Stephens & Balding, [13]). Also, sensitivity analysis can be undertaken to assess the effect of a range of prior probabilities on the TRP.

An interesting property of the TRP (which is related to the false positive report probability introduced by Wacholder *et al*. [10] through TRP = 1 - FPRP) is its direct analogy with the PPV. The TRP can be viewed as the posterior probability of an association which reflects the update of the prior probability through the results of a replication study. In contrast, it is not possible to directly relate the FPRP with a prior probability.

**Figure 1:**
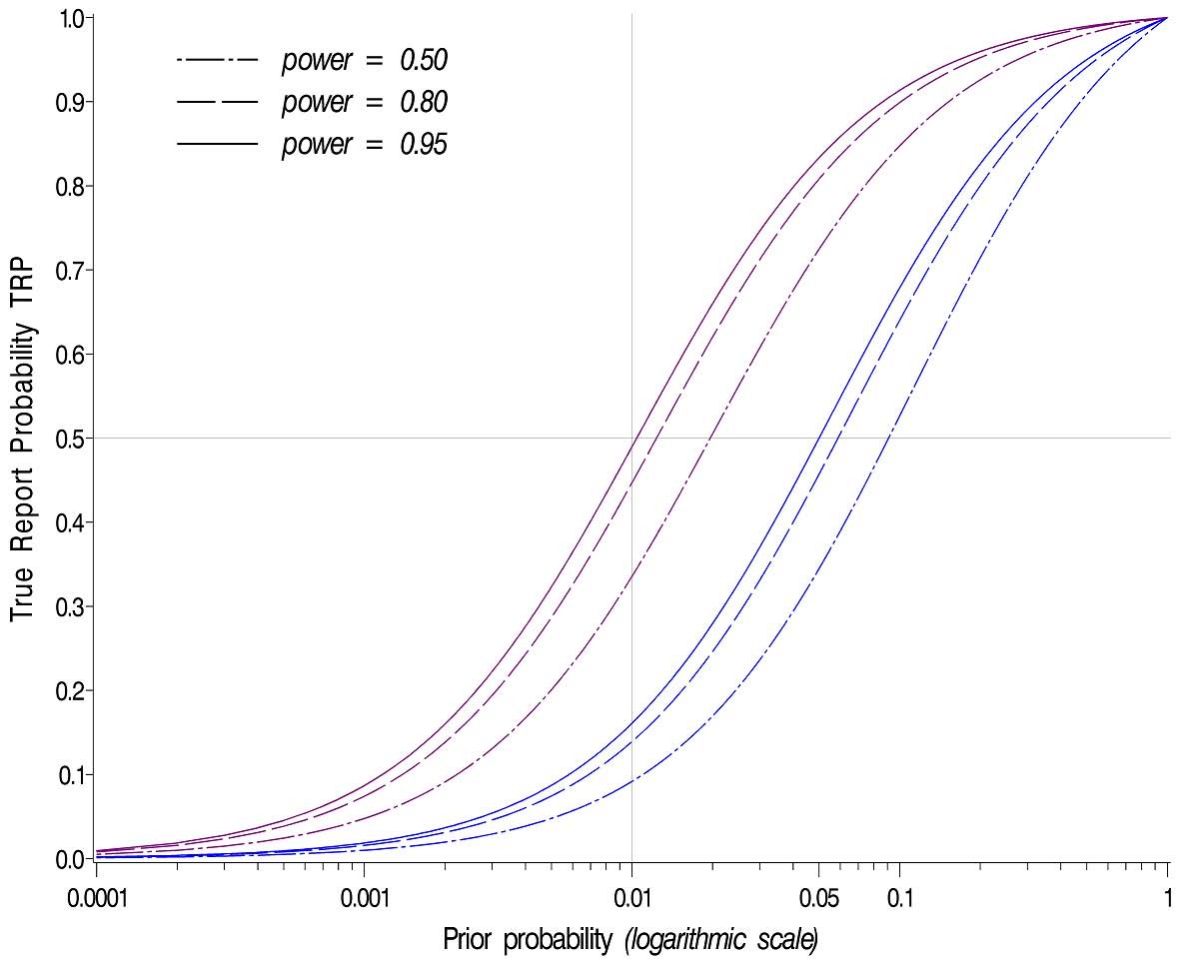
**TRP as a function of the prior probability**. TRP as a function of prior probability (abscissa), α-level (1% and 5%, corresponding to the purple and blue curves, respectively), and power.

Figure 1 shows the TRP as a function of the prior probability of an association π and for several combinations of power and α-level. As can be seen, even in the event of very high power (≈0.95), the TRP remains low when the prior probability of an association is low. For instance, the TRP is below 0.5 when the prior probability is less than 0.01, meaning that less than 50 percent of associations declared as being statistically significant are indeed true associations. A TRP of 0.5 is neither encouraging nor reassuring, as the level of certainty with a significant result corresponds to the outcome probability of tossing a coin.

### Effect of Replication on TRP

In analogy to repeated diagnostic testing, the TRP as defined in (2) can be extended to the case of replication studies, with α and 1 - β having the same values in each study. The TRP obtained after k-1 replication studies is then used as the prior probability for the k^th ^replication study, i.e. the (k + 1)^th ^study.(3)

This formula can be replaced by a simpler formula (see additional file 1: Appendix B for the proof):(4)

It can be seen that in (4) TRP(k+1) only depends on the prior probability π, on α, and on 1-β, but not on the TRP of the previous step, which avoids the need for iterative calculations.

**Figure 2:**
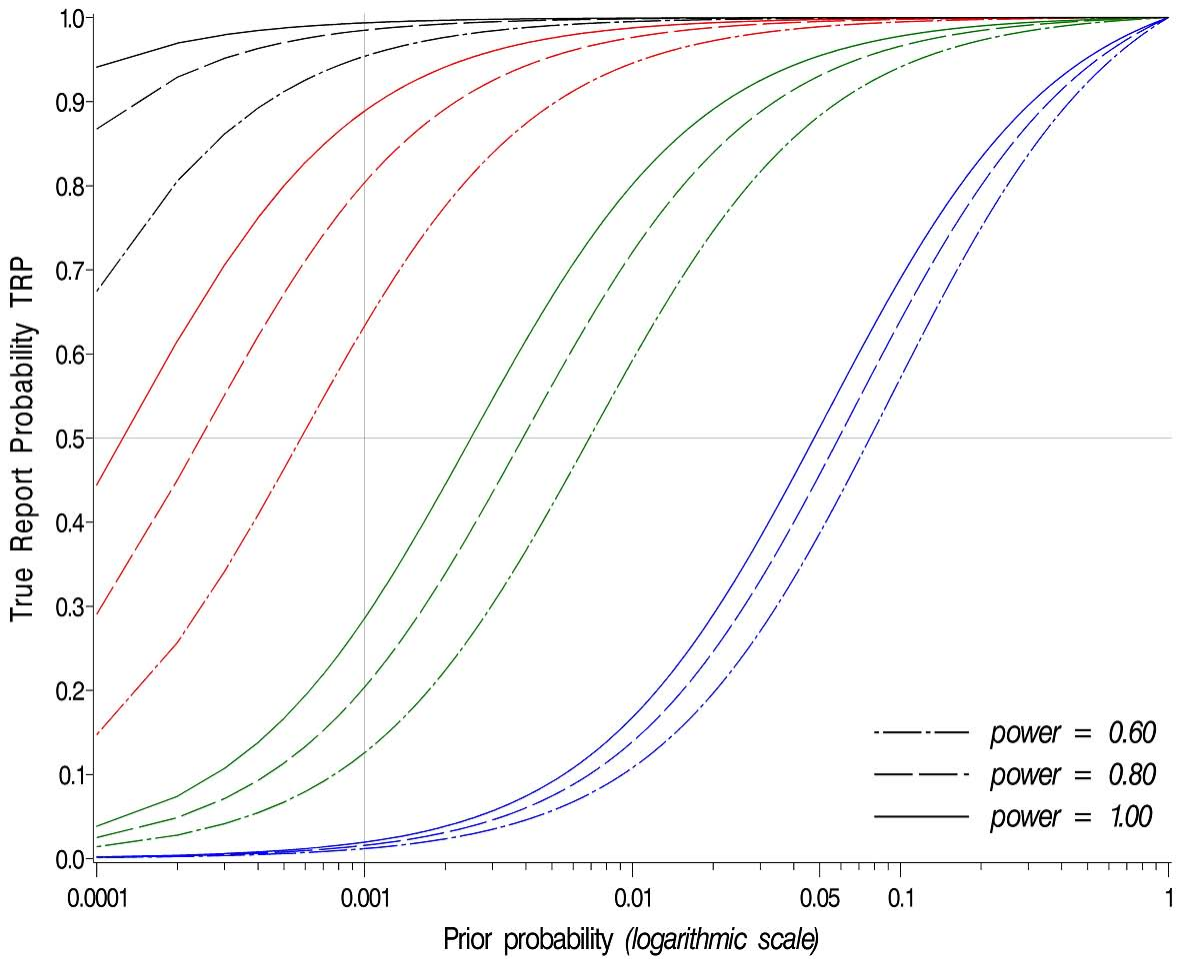
**TRP as a function of the prior probability and number of studies**. TRP as a function of prior probability (abscissa), power, and number of studies (α = 5% in all studies). The three blue, green, red, and black curves represent the case of one, two, three, and four studies, respectively.

Figure 2 represents, for different power levels (identical across all studies of a replication design; α = 0.05 in all studies) the TRP after 1, 2, 3, and 4 studies as a function of the prior probability π of an association. It becomes evident that the TRP cannot exceed 0.5 in the case of only one replication study when the prior probability is below 0.001, even when the power is optimal (i.e. close to unity). In addition, increasing the power exerts only a small effect on the TRP, compared to conducting a replication study, which is reflected in the small elevation of the TRP curve upon increasing the power, as compared to much larger shift in the event of conducting replications. In fact, replication studies are mandatory in order to substantially increase the confidence in the findings, especially when the prior probability is very small. This is of particular relevance in multi-variable genome-wide association research, where often little to no prior knowledge of genetic disease mechanisms is available.

Formula (4) can be used to determine the appropriate research strategy. Given α and π of the initial study, the power and number of replication studies can be determined in order to achieve a desired TRP.

### Finding the Appropriate Research Strategy

The problem is illustrated in Figure 2. When, for example, the prior probability is 0.01 and a TRP of 0.8 is desired, the researcher has two choices: either to conduct three studies with a power of 0.60 each, or two studies with a power of 0.99 each. In both cases, in order to increase the power from 0.60 to 0.99, an increase in the overall (i.e. across studies) sample size is required.

To understand which of the two strategies (i.e. two or three studies) is more efficient, formula (4) can be rearranged:(5)

**Figure 3:**
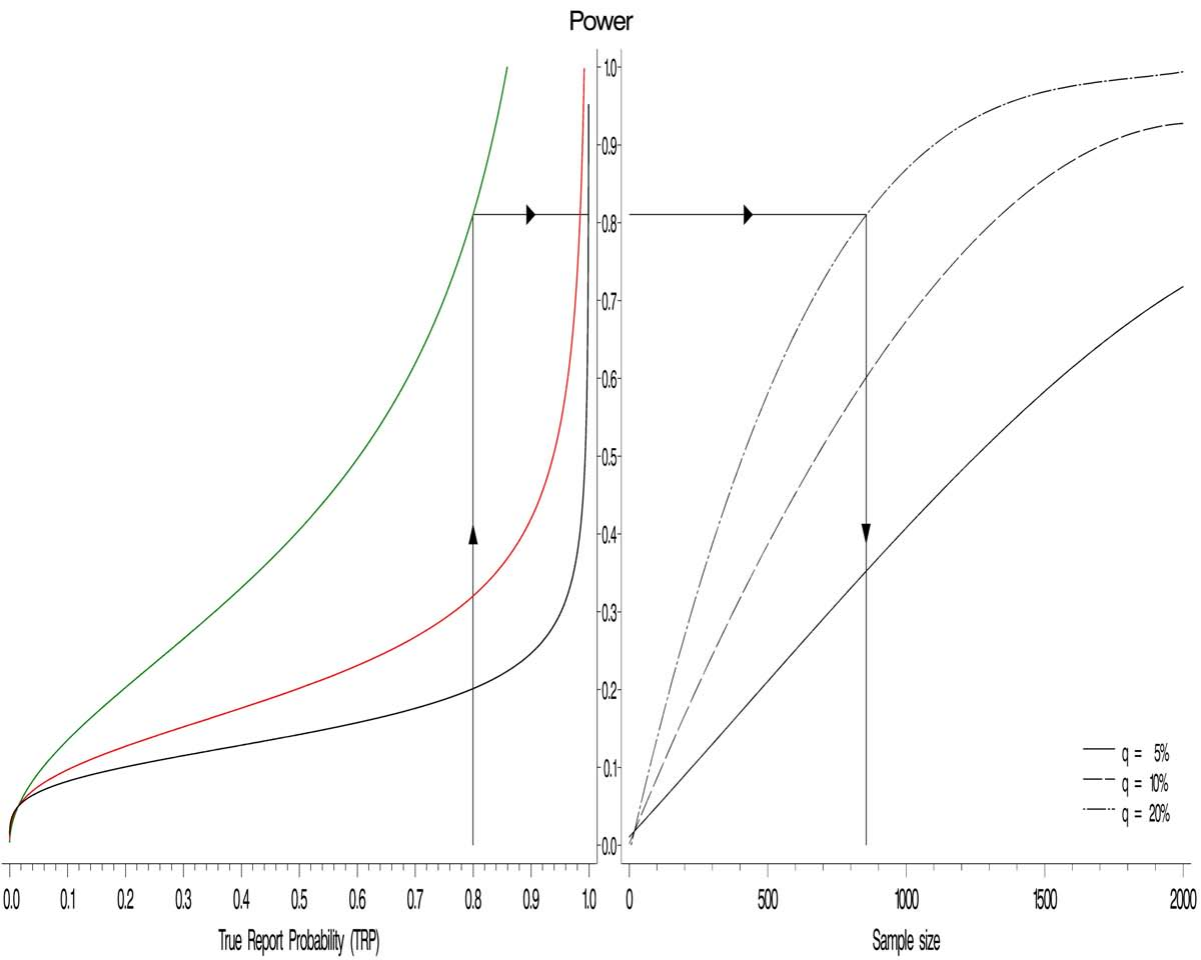
**Relationship between TRP and sample size via power**. Relationships among TRP, power, and sample size for two, three, and four studies (green, red, and black curves, respectively). The prior probability is assumed to be 0.015 (α = 5% in all studies).

In (5), the power of each study is interpreted as a function of the TRP for various values of the prior probability π and number of replications k (note that α is assumed to be constant across the studies). Figure 3 (left panel) illustrates the situation for π = 0.015 and 2, 3, and 4 studies. Both Figure 3 (left panel) and formula (5) can be used to determine the power of each study when the prior probability of an association π and the number of studies are given. Assuming that the TRP should be 0.8, the α-level 0.05 and the prior probability 0.015, it follows that the required power of one individual study must be 0.81, 0.32, or 0.20 if two, three, or four successive studies are performed, respectively.

Since the power largely depends on the sample size and the α-level, it is possible to determine the minimum sample size required to achieve a desired power at a given α-level. To illustrate this relationship, the power curves contained in Figure 3 (right panel) are shown for the study design suggested by Wacholder *et al*. [10] (see Additional file 1: Appendix C for details). The plots illustrate how sample size and the proportion of individuals with both the disease and the genetic variant under investigation (q) are related to power.

The graph can also be used in the reverse manner: given q, it is possible to estimate the required sample size in order to achieve a desired power. For instance, if q equals 0.2 and a power 0.8 is required, the sample size should be at least 820 (corresponding to a power of precisely 0.798). Based on the fact that power is a function of TRP (Figure 3, left panel) and of sample size (Figure 3, right panel), it is possible to establish a link between the final TRP and the required sample size by plotting the two panels of Figure 3 back to back, with power being the common ordinate.

Figure 3 can therefore be used for parsing from the desired TRP (left X-axis) to the required sample size (right X-axis) by following the vertical and horizontal lines through the power axis (and vice versa). By fixing the desired TRP and the number of studies, for instance to TRP = 0.8 and k = 2, it is possible to determine the required power (0.81 in the example) on the power axis and the corresponding sample size for a specific value of q; e.g. for q = 0.2 the sample size would be n = 860.

Similarly, by initially fixing the power to 0.5, the sample size (per study) must be n = 420 and the corresponding TRP will be 0.60 after two studies (0.95 after three studies). By considering different scenarios of power, number of replication studies, and TRP, the replication strategies contained in Table 2 can be compared.

**Table 2:** Replication strategies to achieve a desired TRP (α = 0.05, π = 0.015, q = 0.20).

Number of studies	Sample size per study/Total	Power per study	Final TRP after k studies
2	860/1720	0.81	0.80
	420/840	0.50	0.60
3	260/780	0.32	0.80
	420/1260	0.50	0.95
4	140/560	0.20	0.80

**Figure 4:**
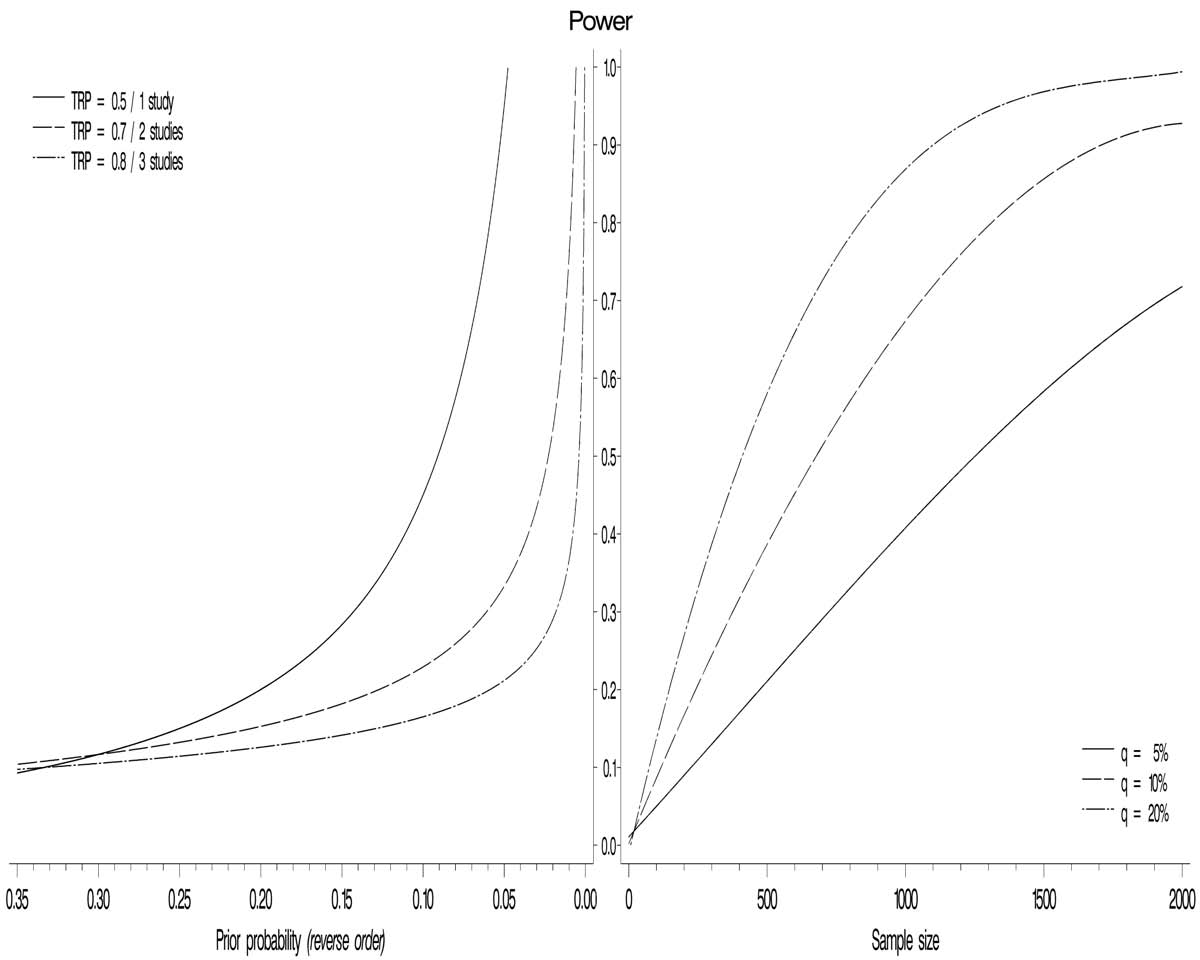
**Relationship between prior probability and sample size via power**. Relationships among prior probability, power, and sample size for combinations of TRPs and numbers of studies (α = 5% in all studies).

To assess the influence of the prior probability, a similar graph can be plotted with the prior probability on the left X-axis and various curves representing TRP levels. Figure 4 displays some combinations of TRP levels and numbers of studies. It is used analogically to Figure 3. The sample size is estimated given a desired TRP and an assumed prior probability of an association.

## Discussion

The concept of TRP was introduced by analogy to the PPV of diagnostic testing and extended to consider the effects of replication studies. The PPV and TRP are about assessing the validity of positive findings and are posterior probabilities. As with the positive predictive value, which can be calculated for a battery of sequentially administered diagnostic tests, calculation of the true report probability can be extended in a straightforward way to research designs involving replication studies. Through this property, the TRP supports the selection of efficient research strategies.

As often when concepts are translated from one domain to another, the analogy between statistical testing as conducted in replicated association studies and diagnostic testing conducted in clinical settings does not hold completely: in sequential diagnostic testing, different testing procedures are applied to the same individuals, while the same (statistical) testing procedures are conducted in different individuals in replication studies. While this difference is a logical consequence of the different domains of diagnostic testing on one hand and statistical testing of associations on the other, the correspondence of the concepts of PPV and TRP is comprehensive, in particular regarding the key aspect of updating available a priori knowledge: as with the PPV, the TRP depends heavily on the prior probability of an association and cannot reach convincing values in one single pass when the prior probability is low, even if the power of the underlying statistical test is high. By extending the TRP concept to replication studies it becomes evident that replication is more efficient than increasing the sample size with regard to raising the validity of positive findings. As joint analysis is more powerful than separate analyses of replication studies (Skol *et al*., [14]), it is a reasonable approach if the main research objective is to discover unknown associations. Likewise, joint analysis may be advantageous for obtaining more precise effect estimates than a smaller replication sample can provide. If, however, avoiding false discoveries is of prime importance, it is necessary to conceive research strategies which plan for replications.

The replication strategy has also been advanced by Zehetmayer *et al*. [15] who "propose multi-stage procedures controlling either the family-wise error rate (FWER) or the false discovery rate (FDR) and derive asymptotically optimal stopping boundaries and sample size allocations (across stages) to maximize the power of the procedure". To avoid further confusion from invalid, especially false-positive reports, it is important that rational research strategies are implemented in association studies (cf. [16]).

Based on the proposed methods, it is possible to estimate the required power/sample size required to achieve a desired TRP, given a prior probability of the association and a certain α-level. The described methodology allows for assessing whether it would be more favourable to perform two, three or more replication studies rather than fewer but larger (and more powerful) studies.

## Conclusions

This paper introduces the concept of true report probability (TRP) by analogy to diagnostic testing. As the concept extends to conducting replication studies, it supports designing effective research strategies. It is shown that replication is more effective in distinguishing spurious from true associations than is increasing the power of individual studies. A framework is provided to support designing the most appropriate research strategy in order to maximise the confidence in "significant" statistical association test results.

## Competing interests

The authors--RW, EK, GV, and GK--are all current employees of Philip Morris Products S.A.

## Authors' contributions

RW initiated the publication, contributed to the writing and finalization of the manuscript. EK derived most of the formulas, did all calculations and contributed to the writing and figures. GV contributed to the writing and created the figures. GK added the analogy of diagnostic testing and the TRP and contributed to the finalization of the manuscript. All authors read and approved the final manuscript.

## Pre-publication history

The pre-publication history for this paper can be accessed here:

http://www.biomedcentral.com/1471-2288/10/47/prepub

## Supplementary Material

Additional file 1 **This PDF (Adobe Acrobat) file contains three appendices**. Appendix A contains the detailed tables related to diagnostic testing and its application to HIV testing. Appendix B contains the proof of the formula for the TRP after k replications. Finally, Appendix C derives a formula that expresses the TRP as a function of the sample size. Click here for file
