# Supplementary material for: Effectiveness of strategies to increase the validity of findings from association studies: size vs. replication
Source: BMC Med Res Methodol. 2010 May 28;10:47. doi: 10.1186/1471-2288-10-47 (PMC2896945; doi:10.1186/1471-2288-10-47)
Supplement: Additional file 1 — This PDF (Adobe Acrobat) file contains three appendices. Appendix A contains the detailed tables related to diagnostic testing and its application to HIV testing. Appendix B contains the proof of the formula for the TRP after k replications. Finally, Appendix C derives a formula that expresses the TRP as a function of the sample size. [file 1471-2288-10-47-S1.PDF]

Appendix A: *Results of Successive Diagnostic Tests*

Table A1 - Diagnostic Test and Disease Status.

Test Result	Disease Status	
	Presence of disease (D+)	Absence of disease (D-)
Positive (T+)	A<br>(true positive)	B<br>(false positive)
Negative (T-)	C<br>(false negative)	D<br>(true negative)

Sensitivity: P(T+ | D+) = A/(A+C)

Specificity: P(T- | D-) = D/(B+D)

PPV: P(D+ | T+) = A/(A+B)

Table A2 - Results of the Elisa Test for HIV.

Assumptions: Sample size = 1’000’000, sensitivity = 0.95, specificity = 0.99, prevalence of the disease = 0.001.

Test Result	Disease Status		
	Presence of disease (D+)	Absence of disease (D-)	Total
Positive (T+)	950	9’990	10’940
Negative (T-)	50	989’010	989’060

PPV = 0.087

Table A3 - Results of the Western Blot Test for HIV.

Assumptions: Sample size = 10’940 (= individuals who tested positive with the Elisa test), sensitivity = 0.95, specificity = 0.99, prevalence = 0.087 (in those who tested positive in the Elisa test).

Test Result	Disease Status		
	Presence of disease (D+)	Absence of disease (D-)	Total
Positive (T+)	902	100	1’002
Negative (T-)	48	9’890	9’938

PPV = 0.90

Appendix B: *Proof of Formula (4)*

The proof is conducted by induction.

Formula (4) holds for k = 0 (see (2)).

Assume that it holds at the kth step, $k \geq 1$, i.e.

$$\mathrm{TRP}(k) = \frac{\pi \cdot (1-\beta)^k}{\pi \cdot (1-\beta)^k + (1-\pi) \cdot \alpha^k}$$

By definition, the TRP obtained after k studies is used as the prior probability for the $(k+1)^{th}$ study, and the samples collected in either study are independent. The TRP after study k + 1, TRP(k+1), is

$$\mathrm{TRP}(k+1) = \frac{\mathrm{TRP}(k) \cdot (1-\beta)}{\mathrm{TRP}(k) \cdot (1-\beta) + \alpha \cdot (1 - \mathrm{TRP}(k))}$$

$$= \frac{(1-\beta) \cdot \dfrac{\pi \cdot (1-\beta)^k}{\pi \cdot (1-\beta)^k + (1-\pi) \cdot \alpha^k}}{(1-\beta) \cdot \dfrac{\pi \cdot (1-\beta)^k}{\pi \cdot (1-\beta)^k + (1-\pi) \cdot \alpha^k} + \alpha \cdot \left(1 - \dfrac{\pi \cdot (1-\beta)^k}{\pi \cdot (1-\beta)^k + (1-\pi) \cdot \alpha^k}\right)}$$

$$= \frac{\dfrac{\pi \cdot (1-\beta)^{k+1}}{\pi \cdot (1-\beta)^k + (1-\pi) \cdot \alpha^k}}{\dfrac{\pi \cdot (1-\beta)^{k+1}}{\pi \cdot (1-\beta)^k + (1-\pi) \cdot \alpha^k} + \alpha \cdot \left(\dfrac{\pi \cdot (1-\beta)^k + (1-\pi) \cdot \alpha^k - \pi \cdot (1-\beta)^k}{\pi \cdot (1-\beta)^k + (1-\pi) \cdot \alpha^k}\right)}$$

$$= \frac{\pi \cdot (1-\beta)^{k+1}}{\pi \cdot (1-\beta)^{k+1} + (1-\pi) \cdot \alpha^{k+1}}$$

In a same manner, the formula for the FPRP after k + 1 studies can be proven:

$$\mathrm{FPRP}(k+1) = \frac{\mathrm{FPRP}(k) \cdot \alpha}{(1 - \mathrm{FPRP}(k)) \cdot (1-\beta) + \mathrm{FPRP}(k) \cdot \alpha}$$

$$= \frac{(1-\pi) \cdot \alpha^{k+1}}{\pi \cdot (1-\beta)^{k+1} + (1-\pi) \cdot \alpha^{k+1}} = 1 - \mathrm{TRP}(k+1)$$

Appendix C: *Relationship between Power and Sample Size*

In a case-control association study (a similar reasoning is possible for other designs), the power can be related to the sample size under similar assumptions as those of Wacholder *et al.* [1]):

- The data are obtained in a case-control association study with N/2 cases and N/2 controls (balanced design).
- The null hypothesis is that the odds ratio is one (H_0: $OR_0 = 1$) and the alternative hypothesis is that the odds ratio is 1.5 (H_1: $OR_1 = 1.5$).
- The odds ratio is assumed to have a log-normal distribution with variance σ^2.
- The type I error rate α is 5 percent
- The proportion of true positives (q) is 5, 10 or 20 percent (proportion of individuals who have the disease and tested positive for the genetic variant under consideration).

The resulting relationships are summarized in Table C1.

Table C1 – Case-Control Study Table.

Test	Cases	Controls
T+	$N \cdot q$	n_{12}
T-	$N/2 - N \cdot q$	$N/2 - n_{12}$

The OR depends only on n_{12}. An estimate of the standard deviation of ln(OR) is given by

$$s = \sqrt{\frac{1}{N \cdot q} + \frac{1}{N/2 - N \cdot q} + \frac{1}{n_{12}} + \frac{1}{N/2 - n_{12}}} \qquad \text{(C1)}$$

The power of the Wald-type hypothesis test is given by

$$1 - \beta = \Phi\{[\ln(OR_1/OR_0)]/\sigma - z_{1-\alpha/2}\}, \qquad \text{(C2)}$$

where Φ is the cumulative distribution function of the standard normal distribution and $z_{1-\alpha/2}$ is the $(1-\alpha/2)$ percentile of the standard normal distribution.

σ is numerically estimated from Table C1, where $\tilde{n}_{12}$ is such that the associated p-value of OR_1/OR_0 equals α, i.e. $\tilde{n}_{12}$ is determined to satisfy the following equation

$$p = 2\left(1 - \Phi\left(\frac{\ln(OR_1/OR_0)}{s}\right)\right) = \alpha\,, \qquad \text{(C3)}$$

where s is determined by (C1). Note that, as n_{12} is discrete, it is generally not possible to find an exact $\tilde{n}_{12}$ such that the associated p-value is exactly equal to α. Instead, $\tilde{n}_{12}$ is determined as the largest integer value among all values that lead to a p-value which is less than or equal to α.

As an example, assume that N = 500, α = 0.05 and q = 0.3 (cf. Table C2).

Table C2 - Distribution of Sample
(Based on the Assumptions of Table C1; N=500, α=0.05 and q=0.3).

Test	Cases	Controls
T+	150	n_{12}
T-	100	$250 - n_{12}$

By n_{12} ranging from 1 to 150 the p-values based on testing the ORs of the resulting 150 tables increase with n_{12}. A portion of this relationship is represented in Figure C1.

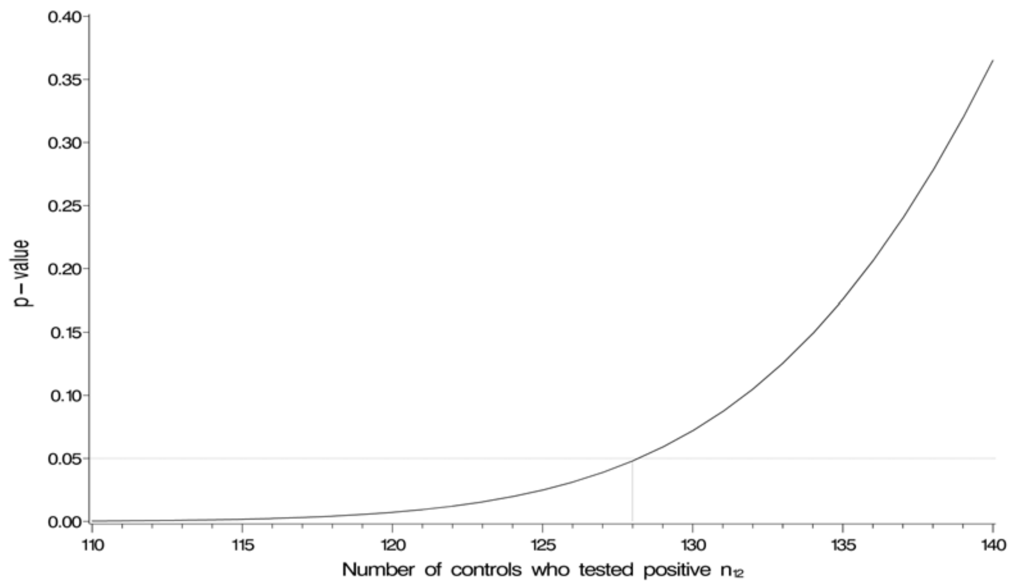

Figure C1 - P-value as a function of the number of controls who tested positive, n_{12} (N = 500, α = 0.05 and q = 0.3).

$\tilde{n}_{12}$ is then the threshold value determined as the maximum over the $1 < n_{12} < 150$ range for which the associated p-value is less than or equal to $\alpha = 0.05$. As can be seen from Figure C1, $\tilde{n}_{12} = 128$. Inserting $\tilde{n}_{12}$ into (C1) allows the estimation of σ:

$$s = \sqrt{\frac{1}{150} + \frac{1}{100} + \frac{1}{128} + \frac{1}{122}} = 0.18 \qquad \text{(C4)}$$

By using the result of (C4) in (C2), one can estimate the corresponding power as being 0.61. This value can be used to estimate the TRP of this study design.

This process is iteratively repeated for all possible sample sizes (from 100 to 10000 by steps of 10), allowing to link various sample sizes to power. Finally, a polynomial curve is applied to smooth the power curve as a function of the sample size. The graph contained in Figure C2 was obtained for $\alpha = 0.05$ and three values of q, 0.2, 0.1, and 0.05.

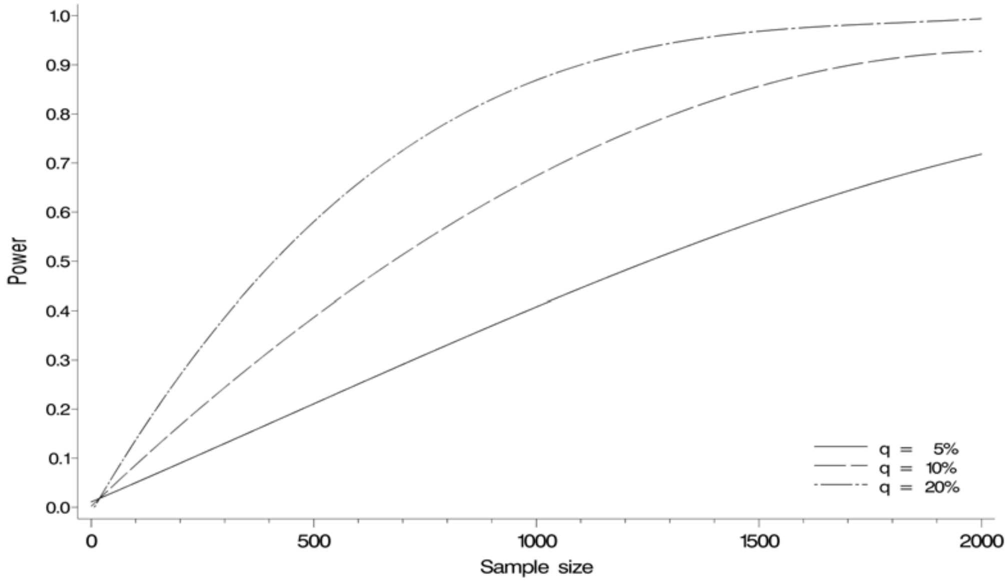

Figure C2 - Power as a function of sample size for various values of q

(α = 0.05 and q = 0.05, 0.1, and 0.2).
